# Structural Vibration Analysis of UAVs Under Ground Engine Test Conditions

**DOI:** 10.3390/s26020583

**Published:** 2026-01-15

**Authors:** Sara Isabel González-Cabrera, Nahum Camacho-Zamora, Sergio-Raul Rojas-Ramirez, Arantxa M. Gonzalez-Aguilar, Marco-Osvaldo Vigueras-Zuniga, Maria Elena Tejeda-del-Cueto

**Affiliations:** 1 Engineering of the Construction and Habitat Faculty, Universidad Veracruzana, Boca del Río 94294, VER, Mexico; zs22024492@estudiantes.uv.mx (S.I.G.-C.); nahumcamachozamora@gmail.com (N.C.-Z.); mvigueras@uv.mx (M.-O.V.-Z.); 2 Office of Graduate Studies, Universidad Aeronáutica en Querétaro, Tequisquiapan 76278, QRO, Mexico; sergio.rojas@unaq.mx; 3 Mechanical Engineering and Naval Sciences Faculty, Universidad Veracruzana, Boca del Río 94294, VER, Mexico; arantgonzalez@uv.mx

**Keywords:** data acquisition system, UAV, vibration analysis

## Abstract

Monitoring mechanical vibration is crucial for ensuring the structural integrity and optimal performance of unmanned aerial vehicles (UAVs). This study introduces a portable and low-cost system that enables integrated acquisition and analysis of UAV vibration data in a single step, using a Raspberry Pi 4B, data acquisition (DAQ) through a MCC128 DAQ HAT card, and six accelerometers positioned at strategic structural points. Ground-based engine tests at 2700 RPM allowed vibration data to be recorded under conditions similar to those of real operation. Data was processed with a Kalman filter, a Hann window function application, and frequency analysis via Fast Fourier Transform (FFT). The first and second wing bending natural frequencies were identified at 12.3 Hz and 17.5 Hz, respectively, as well as a significant component around 23 Hz, which is a subharmonic of the propulsion system excitation frequency near 45 Hz. The results indicate that the highest vibration amplitudes are concentrated at the wingtips and near the engine. The proposed system offers an accessible and flexible alternative to commercial equipment, integrating acquisition, processing, and real-time visualization. Moreover, its implementation facilitates the early detection of structural anomalies and improves the reliability and safety of UAVs.

## 1. Introduction

Unmanned aerial vehicles (UAVs), or remotely piloted aircraft (RPAs), are aircraft capable of remaining airborne without an onboard operator and performing critical activities without risking human life [1,2,3,4]. Currently, UAVs are key tools in aerial photography, agriculture, civil activities, and surveillance [5,6,7,8].

The UAV’s mechanical structure is affected by vibrational disturbances from the operating environment, including structural failures and unexpected vibrations [9,10]. Vibrations can originate from mechanical sources such as rotors, propellers, and actuators. Aerodynamic forces and the UAV’s intrinsic forces also generate vibrations [11,12].

Vibrations are undesired effects that destabilize the system, leading to failures, damage, and reduced flight performance, mainly affecting control and instrumentation systems [10,13]. Moreover, during flight, vibrations can also induce resonance, accelerating the fatigue of aircraft materials [14]. Therefore, monitoring UAV conditions through vibration measurements is essential. It helps assess mechanical integrity and detect anomalies that reveal operational status, improving system efficiency and safety [15,16].

According to E. Ozkat [17], 67% of UAV accidents are linked to mechanical system failures, of which 53% involve the propulsion system. Failures occur mainly in rotors and actuators. These components undergo high stresses during flight, leading to progressive wear and increased probability of structural failures.

From the literature [18,19], UAV vibrations are associated with two sources of propagation: (I) structural vibration, caused by periodic excitation of the engines and transmitted to the aircraft structure, and (II) environmental vibration, generated by the interaction between the structure and the atmosphere airflow. On the other hand, Agrawal et al. [20], indicate that vibrations in aircraft are explained by the phenomenon of Flutter, which corresponds to an unstable condition in which aerodynamic forces excite the natural frequencies of the structure. This type of vibration is particularly undesirable, as it causes performance degradation, structural failure, loss of control, and even complete system failure [21].

Based on previous findings [22,23,24,25,26], UAV failures are divided into two categories: (I) actuator failures, which involve engines, control surfaces, and UAV essential mechanisms. Their malfunction limits maneuverability and diverts the vehicle’s course, increasing accident risk and compromising safety; and (II) sensor failures, which, when exposed to changing flight environment conditions, are likely to cause the loss of the aircraft.

To avoid the failures mentioned above, it is necessary to implement devices and strategies that control the aircraft’s structural response and ensure its integrity. In this context, structural dynamics, through Structural Health Monitoring (SHM), seeks to evaluate the condition, reliability, and integrity of the system using sensor measurements. This approach allows damage to be identified by analyzing changes in modal properties, natural frequencies, modal shapes, and their curvatures. These vibration-based techniques are among the most widely used in SHM due to their physical interpretation of reality [27,28]. Vibration analysis is a non-destructive method widely used in engineering for early damage detection and structural integrity assessment [29]. Ahmed and Nandy [30] describe a vibration-based monitoring system with three fundamental stages: (I) data acquisition, (II) signal processing, and III) diagnosis of the system’s health status. On the other hand, Ground Vibration Test (GVT) is commonly performed on complete structures such as fighter jets, civil aircraft, and unmanned aerial vehicles [31]. The main objective of this vibration test is to identify the model’s dynamic characteristics, including natural frequencies, vibration modes, and damping properties [32].

Zhang et al. [33] classify disturbance detection methods for unmanned aircraft into three main categories: analytical model-based methods rely on mathematical models to represent expected system dynamics and aircraft operation modes. Knowledge-based methods use experience and expertise from specialized expert systems. Finally, signal-processing-based methods focus on extracting features directly from measurement signals and applying tools such as spectral analysis using the FFT.

Several studies have analyzed vibrations in unmanned aerial vehicles. In 2012, Simsiriwong and Sullivan [34] performed a vibration test on the wings of an UAV using sixteen accelerometers installed on a vibration table, obtaining signals of greater amplitude and lower noise levels due to differences in structural stiffness. Meanwhile, in 2013, Lemler and Semke [35] conducted an experimental structural analysis of a small Unmmaned Aerial System (UAS), determining the bending and torsion modes of the wings; data acquisition and processing were performed using ModalVIEW program supported by LABVIEW software (Austin, TX, USA). Similarly, in 2018, Pourpanah et al. [36] developed a monitoring system for early fault detection in unmanned aircraft engines and propellers using an Arduino board, three current sensors, and a three-axis accelerometer.

In 2021, Olejnik et al. [37] performed a structural analysis using data from accelerometers attached to both wings and at asymmetric points, and hardware comprising modal analyzers, vibration exciters (shakers), signal amplifiers, and a computer employing Test.Lab software (Plano, TX, USA). Likewise, in 2022, Ghazali and Rahiman [38] proposed a vibration-based fault detection system using an Arduino UNO microcontroller, four SW420 vibration sensors, an HC05 Bluetooth module for mobile device connectivity, and a 5009 mAh battery. In 2023, Al-Haddad and Jaber [39] designed a high-performance data acquisition system to locate and classify faults, using an STM32H743IIT6 microcontroller, four sensors, and passive components for vibration data processing. Finally, in 2024, Al-Haddad et al. [40] proposed a data processing system based on a DAQ–6009 device, an ADXL335 accelerometer, a laptop, and code developed in LABVIEW software.

In all the studies presented above, the workflow is divided into two stages: (I) data acquisition and (II) reconstruction and analysis of vibration signals. This study proposes the development of a compact, data-acquisition system implemented with a Raspberry Pi 4B, a MCC128 DAQ HAT card, and six ADXL335 accelerometers. Its architecture allows, for the first time, the integration of the two stages (acquisition and analysis of vibration data) into a single process, in contrast to the systems reported in the state of the art. It also has the advantage of being lightweight and flexible, making it easier to install across different configurations and test environments. In addition, it represents an affordable alternative to other vibration-analysis equipment, particularly given its low cost. The scientific contribution comprises the experimental characterization of the UAV’s behavior and the establishment of a measurement methodology applicable to the analysis and validation of unmanned aircraft. Table 1 presents a general comparison between previous studies focused on vibration analysis and the system proposed in this work.

## 2. Materials and Methods

### 2.1. Data–Acquisition System Assembly

The proposed data acquisition system includes a Raspberry Pi 4 Model B (Cambridge, UK), a battery module for the Raspberry Pi 4B, a MCC128 DAQ HAT card (Austin, TX, USA), six ADXL335 (Norwood, MA, USA) accelerometers, an electrical connection harness, a 32 GB micro-SD card, and both female and male DuPont jumper cables.

The Raspberry Pi 4B was used as the processing and storage unit due to its support for multiple programming languages and its expandability via Hardware Attached on Top (HAT) modules. Table 2 presents the technical parameters of the Raspberry Pi 4B according to the manufacturer’s specifications.

The MCC128 DAQ HAT card provides 16–bit resolution and up to 100 kS/s on eight analog inputs; however, in this work, six channels were enabled, each connected to an ADXL355 accelerometer. In addition, the technical specifications of the MCC128 card and the ADXL335 accelerometers are detailed in Table 3 and Table 4, respectively.

**Figure 1:**
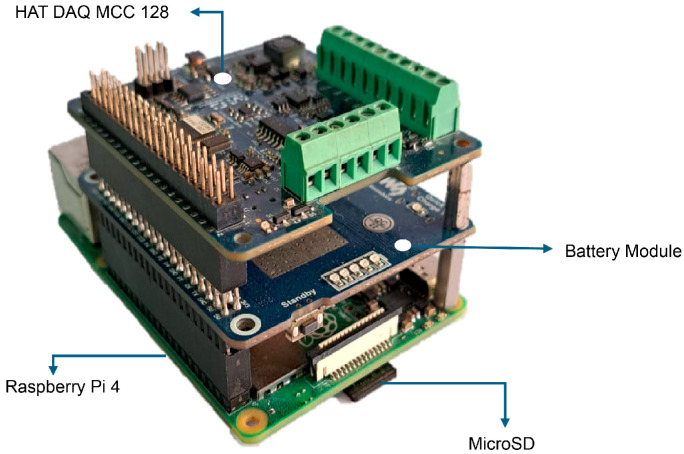
Data–acquisition system configuration.

The ADXL335 accelerometers were configured to record only the Z–axis. This setup enabled the measurement of structural vibrations with low energy consumption and simple integration. The Z–axis was chosen because the most significant vibrations in the UAV structure occur mainly in the direction normal to the surface plane, that is, on the vertical axis. In free configuration tests, Z–axis exhibits the greatest dynamic response to external excitations. Therefore, its analysis is sufficient to characterize the system’s vibratory behavior. Additionally, single-axis acquisition reduces computational resource consumption and simplifies signal processing enhancing Raspberry Pi 4B performance. For implementation, the MCC128 DAQ HAT card and battery module were attached to the Raspberry Pi 4B, forming a compact system, as shown in Figure 1.

**Figure 2:**
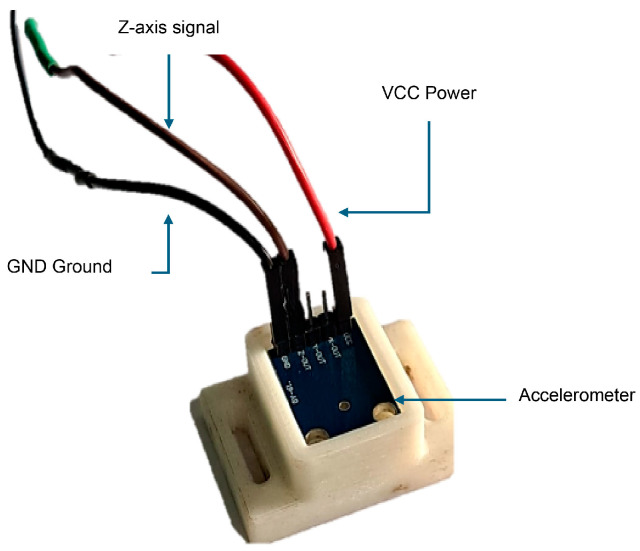
Electrical harness connection to the accelerometer.

Furthermore, sensors were connected via an electrical harness designed to ensure a stable and organized connection, color–coded (red = VCC, black = GND, and brown/white = Z–axis signal). This setup ensures optimal signal transmission and facilitates sensor identification and replacement during testing. Figure 2 shows the connection of an ADXL335 accelerometer installed on a 3D-printed base and connected to the electrical harness using DuPont-type (Shenzhen, China) cables.

Considering all device terminals and their proper assembly, Table 5 presents the final connection diagram for the accelerometers and the data acquisition system, indicating each channel’s pin, function, and cable color.

**Figure 3:**
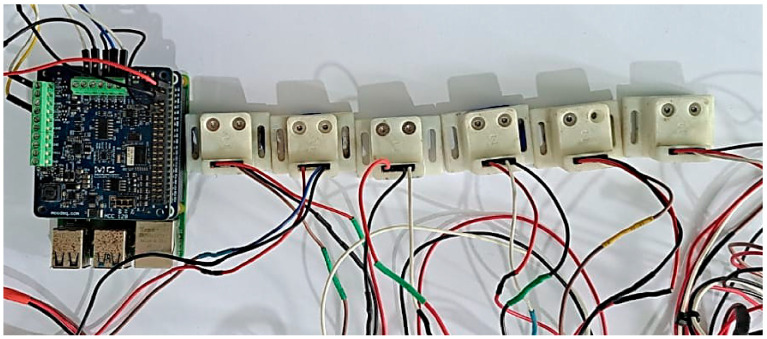
Final configuration of the DAQ hardware.

Figure 3 shows the complete system assembly, including the acquisition card connected to the Raspberry Pi 4B and the six accelerometers properly connected to the electrical distribution harness. This connection facilitates the identification of each channel and ensures proper experimental data collection.

Moreover, Table 6 presents a detailed breakdown of the costs associated with the components of the proposed data acquisition system.

### 2.2. Data Acquisition Program Design

**Figure 4:**
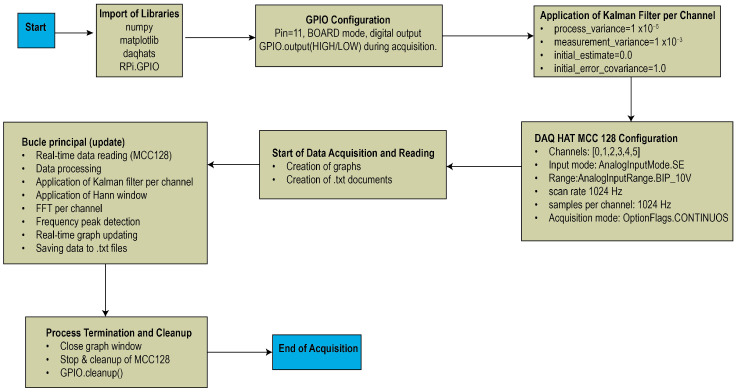
Data acquisition and processing program flowchart.

The procedure implemented for the data acquisition code in this work follows the steps shown in Figure 4. The system was developed using the Python software programming language due to its versatility and the availability of specialized libraries for signal processing, graphical visualization, and communication with acquisition devices. Python software through modules such as NumPy, matplotlib, daqhats, and RPi.GPIO enabled efficient structuring of each stage of the system, from configuring the MCC128 DAQ HAT card to implementing the Kalman Filter and spectral analysis. The initial configuration includes assigning the General-Purpose Input/Output (GPIO) port and connecting to the MCC128 DAQ HAT device, where the acquisition parameters are defined, such as the number of channels, input mode, range, and sampling frequency. Subsequently, the Kalman filter configuration is established, including process and measurement variances, which improve the quality of the recorded signals. During execution, the system continuously acquires data, applies the appropriate filter, and performs spectral analysis using the FFT. Finally, the graphs in the time and frequency domains are updated, and the results are stored in text files for further analysis. The procedure concludes with the cleaning of the system resources and the completion of the acquisition process.

### 2.3. Aircraft Characteristics Identification

The unmanned aerial vehicle is fixed-wing, with a central fuselage, large wingspan wings, control surfaces, and a thrust-vectoring propulsion system. The wings, made of composite materials, reduce weight without compromising stiffness, while the fuselage houses the control, communication, and data transmission electronics. The propulsion system consists of a combustion engine powering a rear-mounted pusher propeller, complemented by a fixed landing gear. These structural characteristics enabled identification of key areas for vibration monitoring, particularly at the wing joints, the central fuselage, and the propulsion system.

#### Selection of Measurement Points for Vibration Analysis

**Figure 5:**
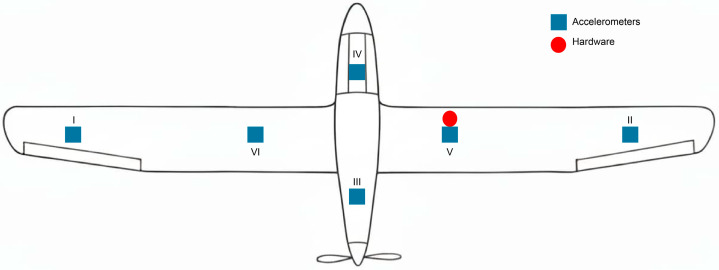
Measurement points diagram on the UAV structure.

Six strategic points were selected on the UAV structure for vibration monitoring, focusing on areas subject to greater structural stress and close to the combustion engine, as well as on critical aerodynamic surfaces. Figure 5 shows the diagram of the established measurement points, while Table 7 details the location of each sensor on the aircraft.

**Figure 6:**
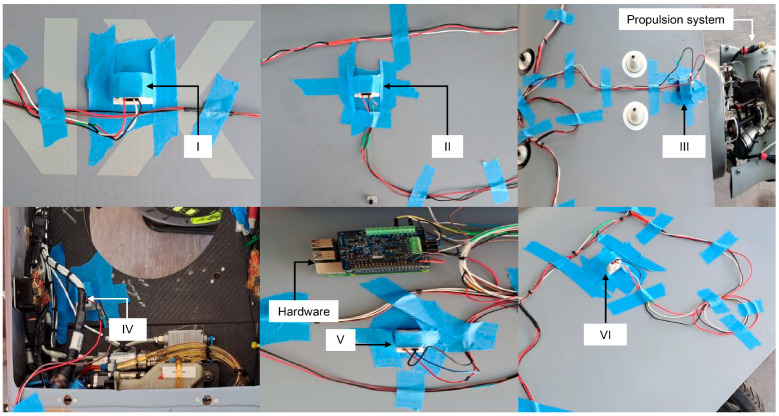
Complete assembly of the data acquisition system on the UAV.

After all connections were established, the system was installed on the aircraft structure to be monitored. For that purpose, the accelerometers were placed at the previously defined measurement points, and the wiring was routed appropriately, and secured with masking tape to prevent interference during data acquisition. Figure 6 shows the physical installation of the sensors and acquisition hardware as detailed in Table 7.

It is important to note that the number and placement of sensors at measurements points may vary with the aircraft’s structural and dynamic characteristics and the analysis objectives. Careful selection of measurement points is essential to obtain data representative of the UAV’s dynamic behavior, considering its geometry, materials, and critical areas subject to aerodynamic stresses.

During the tests, the system recorded the vibration signals and displayed them simultaneously in the time and frequency domains using a graphical interface. At the end of the test, the vibration data was stored in text files, ensuring its availability for further analysis. The records include time-domain signals, with the time values and magnitudes for each channel, as well as frequency-domain results obtained using FFT, with the identified frequencies and their magnitudes recorded.

The aircraft’s intrinsic telemetry indicated an engine speed of 2700 RPM, equivalent to 45 Hz. This telemetry data provides the engine rotation frequency and serves as a reference for identifying whether the structure vibrates in correspondence with this excitation.

## 3. Results

Various experimental tests were conducted to acquire vibration data from the structure of an unmanned aerial vehicle with the engine running. The acquisition hardware was developed, and six accelerometers were distributed at strategic points on the structure and used. Each test lasted 30 min, during which the UAV’s dynamic responses were recorded under real operating conditions. The data obtained enabled identification of the main excitation frequencies, analysis of vibration propagation throughout the structure, and verification of the acquisition system’s proper operation.

**Figure 7:**
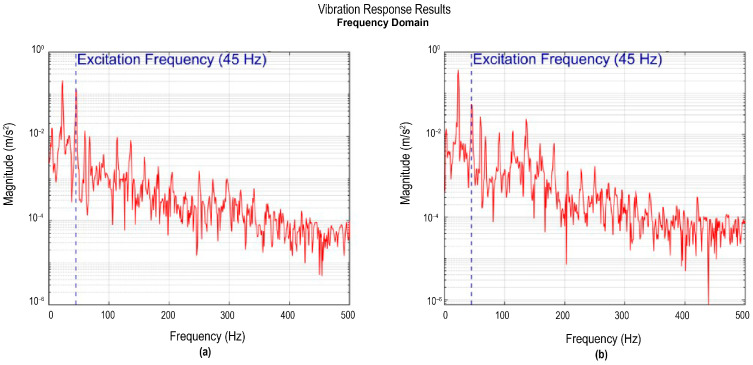
Vibration response recorded by accelerometers I and II: (**a**) signal from accelerometer I in the frequency domain; (**b**) signal from accelerometer II in the frequency domain.

Accelerometers I and II, located at the wingtips, were used to record the regions of greater dynamic amplitude in the system. Figure 7a shows the frequency spectrum corresponding to accelerometer I, where a frequency peak is identified at around 23 Hz with a magnitude of 0.213 m/s^2^. This frequency peak is identified as an engine subharmonic, since it corresponds approximately to half the excitation frequency. The sharp, well-defined peak shape indicates weak damping and a predominant excitation in this mode. Likewise, another component of lesser magnitude is observed at higher frequencies. In particular, a peak appears at 45 Hz with an amplitude of 0.132 m/s^2^, which is associated with the engine’s own excitation.

Figure 7b shows the frequency spectrum corresponding to accelerometer II. Similarly to accelerometer I, a main peak is observed at around 23 Hz, with a magnitude of 0.372 m/s^2^. Despite this slight difference in amplitude compared to accelerometer I, both records confirm the presence of vibrations within the same frequency band. It also has a frequency of 45 Hz with a magnitude of 0.55 m/s^2^, associated with the engine excitation.

The presence of the same dominant frequency at both wingtips validates the modal consistency of the structure. Overall, the responses indicate that the wingtips behave as regions of maximum dynamic response, consistent with modal antinodes. In both frequency spectra (Figure 7), the system telemetry simultaneously recorded the engine’s angular velocity, allowing it to identify that the 45 Hz frequency corresponds to the excitation frequency generated by the engine during the tests. The inclusion of this telemetric reference allows differentiation between frequencies inherent to the structure and frequencies induced by mechanical excitation, providing a more complete context.

**Figure 8:**
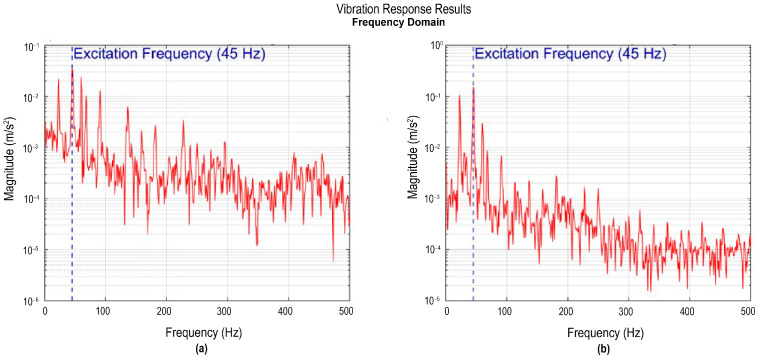
Vibration response recorded by accelerometers III and IV: (**a**) signal from accelerometer III in the frequency domain; (**b**) signal from accelerometer IV in the frequency domain.

Figure 8b illustrates the signal from accelerometer III in the frequency domain. This sensor was located near the engine, at the wing-fuselage joint, so its response is influenced by both the structure’s excitation and the local vibrations generated by the propulsion system. The spectrum indicates the frequency response of accelerometer III, positioned near the engine and thus directly exposed to mechanical vibration. The spectral response shows a dominant peak around 45 Hz with a magnitude of 0.0344 m/s^2^, along with secondary peaks around this fundamental frequency. A lower peak at 23 Hz, with a magnitude of 0.022 m/s^2^, matches a feature in the accelerometer IV spectrum (Figure 8b), indicating a shared structural mode. This pattern indicates that the excitation is not purely sinusoidal but contains a combination of forced frequencies associated with the engine operation. This broader, more complex spectral response suggests an energy-rich vibratory environment, characteristic of areas near active dynamic sources. In addition, the width of the main peak indicates poor damping, which facilitates the transmission of vibrational energy throughout the structure. This broader spectral response, rich in components, reflects a more complex vibratory environment in which forced excitations (produced by the engine) and natural excitations (inherent to the structure) coexist.

Figure 8b shows the corresponding frequency-domain response of accelerometer IV. This sensor, located inside the fuselage, shows a dominant frequency peak at 45 Hz with a magnitude of 0.156 m/s^2^, and two additional components: a peak at 23 Hz with a magnitude of 0.106 m/s^2^, and a frequency peak at 60 Hz with a magnitude of 0.029 m/s^2^. This component is attributed to electrical interference in the environment, commonly associated with the electrical network frequency, and not to a structural mode or mechanical excitation of the system, since its presence is independent of engine operating conditions. The main peak at 45 Hz confirms the presence of engine-induced excitation, which is also observed in accelerometer III, demonstrating dynamic coherence between the two areas of the aircraft and indicating that the vibrational energy of the propulsion system propagates throughout the structure. The component at 23 Hz, also present in the accelerometer III spectrum, corresponds to the first global bending mode. Its presence in both sensors suggests that, in addition to local engine excitations, there is a shared structural contribution related to the system’s modal properties. This comparison shows that sensor location relative to modal conditions and local structural properties significantly influences the recorded amplitude, even when both points are subjected to the same global excitation.

**Figure 9:**
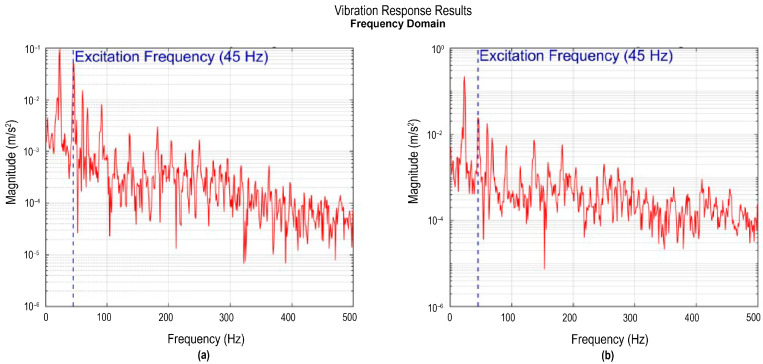
Vibration response recorded by accelerometers V and VI: (**a**) signal from accelerometer V in the frequency domain; (**b**) signal from accelerometer V in the frequency domain.

Accelerometers V and VI were installed in the middle sections of each wing, specifically between the fuselage and the wingtip. Figure 9a shows the corresponding spectrum of sensor V, where a frequency peak of 23 Hz with a magnitude of 0.097 m/s^2^ is visible, along with several higher frequencies with small magnitudes, such as a frequency of 45 Hz with a magnitude of 0.052 m/s^2^ and a peak of 60 Hz with a magnitude of 0.015 m/s^2^. The simultaneous presence of these frequencies reveals the coexistence of natural wing vibration modes, primarily the first bending mode at 23 Hz, with frequencies forced by the engine.

Figure 9b illustrates the frequency spectrum of the accelerometer VI, where a peak at 23 Hz with a magnitude of 0.222 m/s^2^ is observed, corresponding to the first flexural mode of the wing. In addition, peaks at 45 Hz and 60 Hz are observed, although with lower magnitudes than the main peak. The coincidence of the dominant frequency between the accelerometers confirms the presence of a common structural mode in both wings, characteristic of an aircraft’s symmetrical behavior. In aggregate, the responses of accelerometers V and VI exhibit that the mid-wing areas act as dynamic transition regions, where stiffness and vibrational energy transmission are balanced. In addition, a 60 Hz component was detected in both sensors, associated with environmental electrical interference, commonly at the frequency of the electrical grid.

In summary, the experimental results show a 23 Hz measurement across all accelerometers, which correspond to the structure’s fundamental vibration mode. This mode involves the overall motion of the system; therefore, its response propagates across the entire surface of the structure and is independent of the sensor’s positions. Likewise, a second frequency of approximately 45 Hz is identified, associated with a higher flexural mode. The simultaneous appearance of both components confirms that, during excitation, the structure responded with a combination of modes, with the first dominating the overall behavior and the second contributing local variations in the dynamic response. It should be noted that the 45 Hz frequency coincides with the engine speed recorded by ground-based telemetry, indicating the presence of an additional forced component in the aircraft’s vibration response.

**Figure 10:**
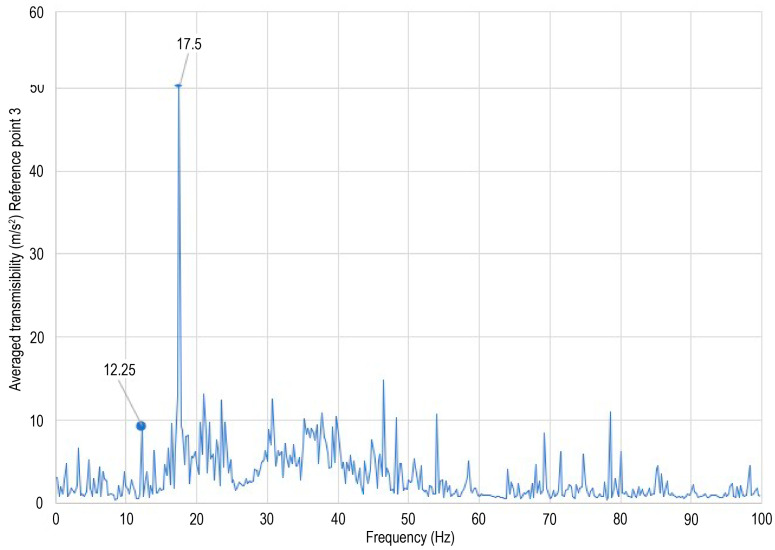
Averaged transmissibility.

**Figure 11:**
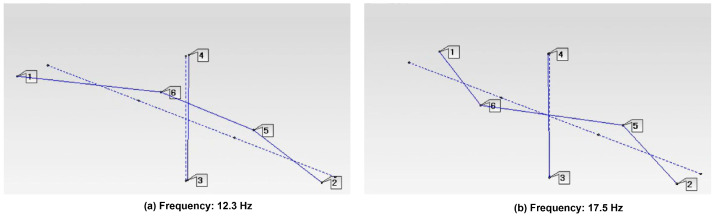
Operational modal shapes.

Ground vibration tests were performed on the unmanned aerial vehicle (UAV), and vibration responses were measured at six distinct structural locations. Transmissibility functions were computed using the response at measurement point 3 as the reference, and the average transmissibility is presented in Figure 10. The analysis corresponds to an operational modal analysis approach, since response-to-response relationships were employed. The first and second wing bending natural frequencies were identified at 12.3 Hz and 17.5 Hz, respectively, as shown in Figure 11. No peak is observed at 60 Hz in Figure 10, confirming that this frequency component is associated with electrical noise rather than structural dynamics. Although additional peaks appear at frequencies above 17.5 Hz, they are not discussed further, as the limited number of measurement points does not allow reliable identification of higher-order or more complex mode shapes. Furthermore, no peak is observed at 45 Hz, indicating that the excitation frequency does not influence the transmissibility-based analysis.

The identified natural frequencies are consistent with previously reported experimental and numerical studies on UAV wing structures, where first bending modes are typically found in the range of 7–15 Hz, depending on wing geometry, material properties, and test configuration [41,42].

### Analysis Results

In this work, a portable data-acquisition and processing system was developed for analyzing vibrations in the structure of a UAV, using a Raspberry Pi 4b, a MCC128 DAQ HAT card, and six accelerometers. The objective was to identify the main excitation frequencies, analyze vibration propagation, and evaluate the performance of the acquisition system under real operating conditions with the engine running. During the experimental test, the UAV’s dynamic responses were recorded in the time and frequency domains.

From the acquisition system results, it can be concluded that

- The acquisition system maintained stable performance throughout testing, consistently recording signals from all six accelerometers under real operating conditions.
- The data obtained will support model training to identify deviations in aircraft structure behavior from nominal parameters.
- The Python-based acquisition code effectively managed data collection, real-time graphical representation, and frequency spectrum calculation. It enabled precise signal recording and visualization, confirming robust communication between hardware devices and digital signal processing.
- Accelerometers I and II, placed at the wingtips, confirmed these locations as modal antinodes, exhibiting maximum dynamic amplitude and reduced structural stiffness.
- Accelerometers III and IV, situated near the engine and within the fuselage, responded mainly to vibrations from mechanical excitations of the propulsion system, with dominant components at 23 Hz and 45 Hz, indicating effective vibrational energy transmission to the fuselage.
- The fuselage functioned as a structural resonance chamber, amplifying vibratory components linked to its natural frequencies and verifying its integral role in vibrational energy transmission.
- Accelerometers V and VI, positioned in the mid-wing areas, recorded intermediate and stable amplitudes. These regions serve as dynamic transitions balancing structural stiffness and flexibility.
- All accelerometers detected a consistent 23 Hz frequency, confirming this as the fundamental mode of the structural system.
- Coincidence between the 45 Hz frequency and engine speed recorded by ground-based telemetry confirms that frequency arises from propulsion system forces.
- Dominant frequencies across all sensors reinforce the UAV’s modal coherence and the efficient propagation of vibrational energy throughout the structure. This provides reliable evidence of symmetrical structural behavior consistent with the aircraft’s mass and stiffness distribution.
- The lightweight data acquisition and processing system can be onboarded for real-time flight analysis or used during ground structural assessments of unmanned aircraft.

This research demonstrates that mechanical vibration analysis can be performed on the ground for a UAV’s structure using data acquisition system. This approach presents an accessible alternative to higher-cost commercial equipment, with the advantage of being adaptable to future studies, both under control and in real operational conditions.

## 4. Conclusions

In this work, a portable data-acquisition and processing system was developed and validated for analyzing vibrations in the structure of an UAV, using a Raspberry Pi 4B, an MCC128 card, and six accelerometers. Experimental test conducted with the engine in operation demonstrated the system’s stable, reliable performance under real operating conditions. The first and second wing bending natural frequencies were identified at 12.3 Hz and 17.5 Hz, respectively, as well as a significant component around 23 Hz, which is a subharmonic of the propulsion system excitation frequency near 45 Hz. The spatial distribution of the responses revealed modal antinodes at the wing tips, amplification in the fuselage, and dynamic transition behavior in the middle regions of the wing, consistent with a symmetrical structural configuration. Overall, the proposed system is a lightweight, flexible, and low-cost alternative to commercial solutions, with potential applications in ground-based structural evaluations and future real-time vibration analyses during flight.

## Figures and Tables

**Table 1:** General comparison of previous vibration analysis studies and the proposed system [33,34,35,36,37,38,39,40].

Technical Aspect	Literature Approach	Technical Gap Identified	Key Parameters	Contribution of This Work
Test conditions	Laboratory test or numerical simulations	Lack of realistic excitation	Engine speed	Ground test with engine operating
Instrumentation	Acquisition system with industrial-grade piezoelectric accelerometers	High cost and low portability	Weight and cost	Compact, scalable, and low-cost system
Number and location of sensors	Limited settings	Partial structural coverage	Spatial distribution	Sensors placed in critical structural areas
Signal processing	Offline FFT or basic filtering	Offline signal processing	Monitoring capability	Real-time filtering and spectral analysis
System integration	Acquisition and analysis by separate stages	Fragmented workflow	System architecture	Acquisition and analysis integration
Application on real UAVs	Test-bench-oriented systems	Limited implementation in small aircraft	Size and energy consumption	Applicable on UAVs during ground tests

**Table 2:** Features of the Raspberry Pi 4B platform.

Parameter	Specifications	Observations
Platform	Raspberry Pi 4 Model B	Embedded computer
Operating system	Raspberry Pi Os (Linux)	32-bits
Programming language	Python	Version 3.11.12
Main libraries	Numpy1.24.2, SciPy 1.10.1, Matplotlib 3.6.3, daqhats 1.5.0.0	For acquisition and analysis
RAM memory	4 GB	Suitable for data acquisition and processing
Power supply	5 V	Via charger or battery module
Communication interface	SPI	Communication with MCC128

**Table 3:** Features of the MCC128 DAQ HAT device.

Parameter	Configuration	Observations
Model	MCC128 DAQ HAT	Measurement Computing
Resolution	16-bits	Analog-to-digital converter
Number of channels	8	Six channels were used
Input model	Single-ended	Differential mode was not used
Input range	±10 V	Configuration used
Sample frequency	1024	Samples were taken by channel
Type of acquisition	Multiplexed	No simultaneous acquisition
Channel synchronization	Not simultaneous	Multiplexer time not specified by manufacturer
Interfax communication	SPI	Via Raspberry Pi

**Table 4:** Operating parameters of the ADXL335 accelerometers.

Parameter	Configuration	Observations
Model	ADXL335	MEMS accelerometer
Number of axes	3	Only the *z*-axis was used
Measurement range	±3 G	According to data sheet
Mass	4 g	The mass of the sensor and the case was considered
Nominal sensitivity	300 Mv/g	Used to convert voltage to acceleration
Supply voltage	3.3 V	Compatible with Raspberry Pi
Output type	Analog	Output voltage proportional to acceleration
Frequency band	Up to 1600 Hz (axis X/Y) and 550 Hz (axis Z)	Restricted by internal filter
Calibration	Not performed	Nominal sensitivity was used

**Table 5:** Final accelerometer connections to the data acquisition system.

PIN	Description	DuPont Color
GPIO2	VCC harness connection	Red
GPIO9	GND harness connection	Black
CH0	Accelerometer I	White
CH1	Accelerometer II	Brown–Green
CH2	Accelerometer III	White–Blue
CH3	Accelerometer IV	Brown–Yellow
CH4	Accelerometer V	Brown–Blue
CH5	Accelerometer VI	White–Yellow

**Table 6:** Cost of the proposed data acquisition system.

Component	Model	Quantity	Unit Cost *	Total Cost *
Embedded computer	Raspberry Pi 4 Model B	1	MXN 2100.00	MXN 2100.00
Acquisition card	MCC128 DAQ HAT	1	MXN 5400.00	MXN 5400.00
Accelerometers	ADXL335	6	MXN 150.00	MXN 900.00
Power supply	USB charger cable	1	MXN 389.00	MXN 350.00
Battery module	Battery module for Raspberry Pi	1	MXN 695.00	MXN 695.00
Micro SD	ADATA 32 GB	1	MXN120.00	MXN 120.00
AWG cable	Red, black, white, and brown #18-gauge cable	24	MXN 5.00	MXN 120.00
Total cost				MXN 9685.00

* Mexican pesos (MXN).

**Table 7:** Accelerometer placement on the UAV structure.

Accelerometer	Description
I	Left wingtip
II	Right wingtip
III	Center wing box
IV	Avionics bay
V	It was situated 50 cm from accelerometer II, and the acquisition system was also fixed at this point, serving as a control point.
VI	It was placed 50 cm from accelerometer I.

## Data Availability

The original code data developed for this work is openly available in GitHub repository at https://github.com/SaraaGlz/uav-vibration-daq (accessed on 9 January 2025).

## Abbreviations

The following abbreviations are used in this manuscript:

DAQ	Data Acquisition
FFT	Fast Fourier Transform
GPIO	General-Purpose Input/Output
GVT	Ground Vibration Test
HAT	Hardware Attached on Top
RPAs	Remotely Piloted Aircraft
SHM	Structural Health Monitoring
SPI	Serial Peripheral Interface
UAS	Unmanned Aerial System
UAV	Unmanned Aerial Vehicle
